# Supplementary material for: Thermal Cycling-Hyperthermia Attenuates Rotenone-Induced Cell Injury in SH-SY5Y Cells Through Heat-Activated Mechanisms
Source: Int J Mol Sci. 2025 Jul 11;26(14):6671. doi: 10.3390/ijms26146671 (PMC12294576; doi:10.3390/ijms26146671)
Supplement: Supplementary file 1 [file ijms-26-06671-s001.zip › ijms-3661594-supplementary.pdf]

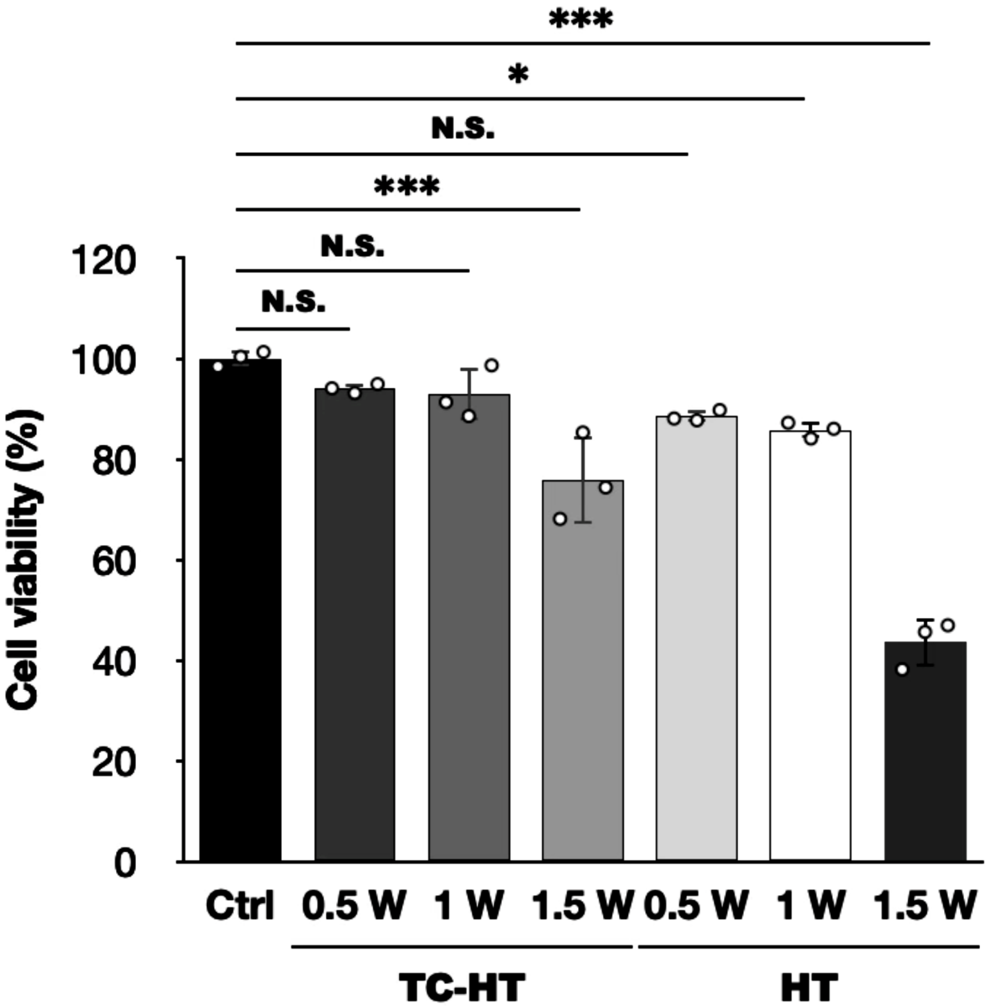

Supplementary Figure S1. Neurotoxicity of TC-HT and HT at various intensities used alone on SH-SY5Y cells. CCK-8 assay was conducted to determine the viabilities of SH-SY5Y cells following TC-HT and HT treatments alone with different US intensities. The viabilities were measured 24 h after TC-HT and HT treatment (*F*-value = 56.8473). Data were presented as the mean ± standard deviation in triplicate. Significance levels between indicated groups are denoted as *P < 0.05 and ***P < 0.001, while non-significant differences are indicated as N.S.

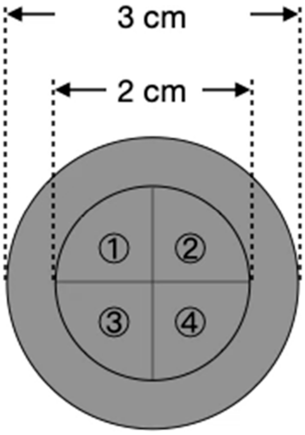

Position	①	②	③	④
Temperature (℃)	40.2	40.3	39.9	40.1

Supplementary Figure S2. Temperature fluctuation measured at multiple positions within the central region (2 cm diameter) of the planar ultrasound transducer (3 cm diameter). Four positions were monitored to assess potential temperature fluctuations across the area where most cells were located.
